# The effect of average temperature on suicide rates in five urban California counties, 1999–⁠2019: an ecological time series analysis

**DOI:** 10.1186/s12889-021-11001-6

**Published:** 2021-05-25

**Authors:** Sierra Cheng, Rebecca Plouffe, Stephanie M. Nanos, Mavra Qamar, David N. Fisman, Jean-Paul R. Soucy

**Affiliations:** Dalla Lana School of Public Health, University of Toronto, 155 College St, Toronto, Ontario M5T 3M7 Canada

**Keywords:** Suicide, Suicide prevention, Climate change, Epidemiology

## Abstract

**Background:**

Suicide is among the top 10 leading causes of premature morality in the United States and its rates continue to increase. Thus, its prevention has become a salient public health responsibility. Risk factors of suicide transcend the individual and societal level as risk can increase based on climatic variables. The purpose of the present study is to evaluate the association between average temperature and suicide rates in the five most populous counties in California using mortality data from 1999 to 2019.

**Methods:**

Monthly counts of death by suicide for the five counties of interest were obtained from CDC WONDER. Monthly average, maximum, and minimum temperature were obtained from nCLIMDIV for the same time period. We modelled the association of each temperature variable with suicide rate using negative binomial generalized additive models accounting for the county-specific annual trend and monthly seasonality.

**Results:**

There were over 38,000 deaths by suicide in California’s five most populous counties between 1999 and 2019. An increase in average temperature of 1 °C corresponded to a 0.82% increase in suicide rate (IRR = 1.0082 per °C; 95% CI = 1.0025–1.0140). Estimated coefficients for maximum temperature (IRR = 1.0069 per °C; 95% CI = 1.0021–1.0117) and minimum temperature (IRR = 1.0088 per °C; 95% CI = 1.0023–1.0153) were similar.

**Conclusion:**

This study adds to a growing body of evidence supporting a causal effect of elevated temperature on suicide. Further investigation into environmental causes of suicide, as well as the biological and societal contexts mediating these relationships, is critical for the development and implementation of new public health interventions to reduce the incidence of suicide, particularly in the face increasing temperatures due to climate change.

**Supplementary Information:**

The online version contains supplementary material available at 10.1186/s12889-021-11001-6.

## Background

Suicide is defined as the act of deliberately ending one’s own life [1]. Suicide is among the top 10 leading causes of premature morality in the United States and is projected to increase in all racial and ethnic groups [2, 3]. Thus, its prevention has become a salient public health responsibility. Suicide is a complex psychopathological phenomenon driven by biological variables, psychological variables, and interactions between individuals and their social environment [4, 5]. Risk factors for suicide transcend the individual and societal level and may include environmental variables such as climate. The effect of ambient temperature on suicide is an important issue to address, particularly with the rise in global temperatures due to climate change [6].

The association of weather variables and human health date back to Hippocrates’ assertion that cold and warm winds can affect physical and psychological health [4, 7]. Contemporary analyses have found an influence of meteorological patterns on human conditions and behaviours including migraine, ischemic stroke, multiple sclerosis, coronary disease, asthma, mortality and suicide [7]. However, the biological mechanisms linking temperature and suicide are not well understood [6, 8]. Understanding the effect of climate on human behaviour is challenging due to the inherent complexity of suicide and environmental variables [4, 7]. No individual suicide can be attributed to a single event and can be compounded by the number of risk factors a person has [4]. Nonetheless, there is a large body of evidence indicating that weather variables can further exacerbate the risk of suicide but the relationship remains inadequately quantified [9, 10].

Heterogeneity in study methods, analysis, and social conditions of different geographic locations may contribute to under- or overestimating the health effects of increased ambient temperature in previous research and make direct comparisons difficult [7, 11, 12]. Previous studies evaluating the association between suicide and temperature have been conducted in nations including India, Japan, New Zealand, Spain, England, and the United States [4, 6, 8, 13]. A systematic review found 15 of the 17 studies examining the association between suicide and temperature to have a positive and significant association [11]. Page and colleagues found a small yet robust impact of temperature on suicide in England and Wales [14]. Furthermore, a large case-crossover study conducted in Spain found an increase in deaths due to suicide associated with an increase in extremely hot ambient temperatures [15]. While aforementioned studies generally report a positive association, according to Williams and colleagues [6], studies concerned with temporal variation in temperature and suicide tend to find a positive association while those concerned with geographical variations tend to find a negative relationship. Longitudinal analyses including multiple locations and many years of consistently measured data at a fine spatial and temporal resolution offer the best chance at accurately identifying the causal effect of temperature on observed patterns of suicide.

California is the most populous state in the United States and is home to over 39 million residents. Notably, suicide has increased in California in recent decades, even after accounting for changes to the population structure over time [16]. Suicide is the second leading cause of death among Californians aged 15–24 years old [17]. As a fairly progressive state, we expect recent suicide mortality data from California to be less subject to underreporting. California has a Mediterranean climate with mild, rainy winters and hot, dry summers combined with varying geography including shorelines, mountains and deserts [18]. The persistent and destructive Californian wildfires in combination with the impacts of increasing temperatures, rising sea levels, ozone depletion, and changing precipitation patterns make California exceptionally vulnerable to the effects of climate change [18]. Given these facts, we believe California to be an important target for research to elucidate the potential effects of increasing temperatures on mortality from suicide.

In this analysis, we aim to characterize the association between temperature and suicide rates in the five most populous counties in California using a time series analysis of monthly mortality and climatic data from 1999 to 2019. To circumvent the issue of spurious association, we first account for the seasonality and annual trend of suicide rates in each county and investigate the impact of short-term variation in temperature [19]. We hypothesized that positive, short-term changes in average temperature (C^o^) would lead to an increase in the incidence in suicide in these five urban California counties.

## Methods

### Data sources

We used publicly available suicide, climate, population, and macroenomic data. Monthly counts of mortality classified as intentional self harm (ICD-10 codes U03, X60–X84, Y87.0) were obtained for all California counties from January 1999 to December 2019 from the Underlying Cause of Death database of CDC WONDER [20]. This database includes mortality data produced by the National Center for Health Statistics [21]. Monthly average, maximum, and minimum temperature data were obtained from the National Oceanic and Atmospheric Administration’s nCLIMDIV database for the same time span (date of data release: 2021-02-04) [22]. Mid-year population estimates for each California county for 1999–2019 were acquired from the U.S. Population Data - 1969-2019 database maintained by the National Cancer Institute’s Surveillance Epidemiology, and End Results Program [23]. Finally, monthly county-level unemployment rates were acquired from the Local Area Unemployment Statistics database maintained by the State of California’s Employment Development Department [24]. Seasonally unadjusted values were used, as seasonally adjusted employment rates were not available at the county level.

### Data cleaning

Monthly counts in the Underlying Cause of Death database are suppressed if they represent fewer than 10 persons (0–⁠9). Since most California counties had fewer than 10 deaths attributable to suicide in each month, most county-level data were suppressed. We limited the analysis to the five counties with fewer than 10% suppressed monthly observations. Counties included: Los Angeles County (0% suppressed), San Diego County (0% suppressed), Orange County (1.19% suppressed), Riverside County (5.59% suppressed), and San Bernardino County (7.14% suppressed). These correspond to the five most populous counties in California. Temperature data were converted from °F to °C according to scientific convention.

### Statistical analysis

We modelled monthly deaths by suicide in five urban California counties using generalized additive models as implemented in the “mgcv” package using R version 4.0.3 [25–27]. Both Poisson and negative binomial models were considered, using a log-link and a county’s mid-year population as the offset. To capture each county’s intercept and annual temporal trend, we used county-specific thin plate regression splines (estimating a single shared smoothness parameter for all splines). We also used a categorical variable for month to account for seasonality [28]. Monthly counts suppressed due to small cell sizes (0–9) were treated as missing values. All model comparison was performed using Akaike information criterion (AIC), with lower values indicating better relative goodness of fit and ΔAIC > 2 indicating substantially better evidence for the model [29, 30].

For each model formulation, we fit both a Poisson model (assuming equal mean and variance of the conditional distribution of the outcome) and a negative binomial model (variance can exceed the mean). First, we fit a baseline model including only the county-specific annual trend and the categorical variable for month. For the primary model, we added a continuous term for monthly average temperature to the baseline model. The exponentiated coefficient for this term corresponds to the incidence rate ratio (IRR) of suicide given a 1 °C increase in average temperature, holding all else constant. Additionally, we fit models substituting monthly average temperature with monthly maximum temperature and monthly minimum temperature. In total, eight models were fit.

### Model checking

The residual distribution of each model was assessed using a quantile-quantile (Q-Q) plot of the deviance residuals. The presence of autocorrelation in the residual time series was assessed using autocorrelation plots stratified by county. If residual autocorrelation is present, an autoregressive correlation structure should be used. However, in many epidemiological applications, residual autocorrelation is eliminated by accounting for other variables, particularly seasonality [19, 31].

### Sensitivity analysis

As a sensitivity analysis for treating suppressed values as missing, we re-ran a subset of the models after imputing values for the suppressed counts. Traditional methods for imputing missing values in a time series (such as cubic interpolation or Kalman filtering) would be of limited use because suppressed values were, by definition, smaller than all observed values in the time series. Instead, we randomly selected values between 0 and 9 from a discrete uniform distribution.

In another sensitivity analysis, we fit additional models adding the county-level unemployment rate as a variable to our best-fit model from the primary analysis. We fit 7 total models using 7 different lags for the unemployment rate (0–6 months) and assessed whether the estimated coefficient for the main variable of interest (temperature) changed compared to the best-fit model.

## Results

**Fig. 1:**
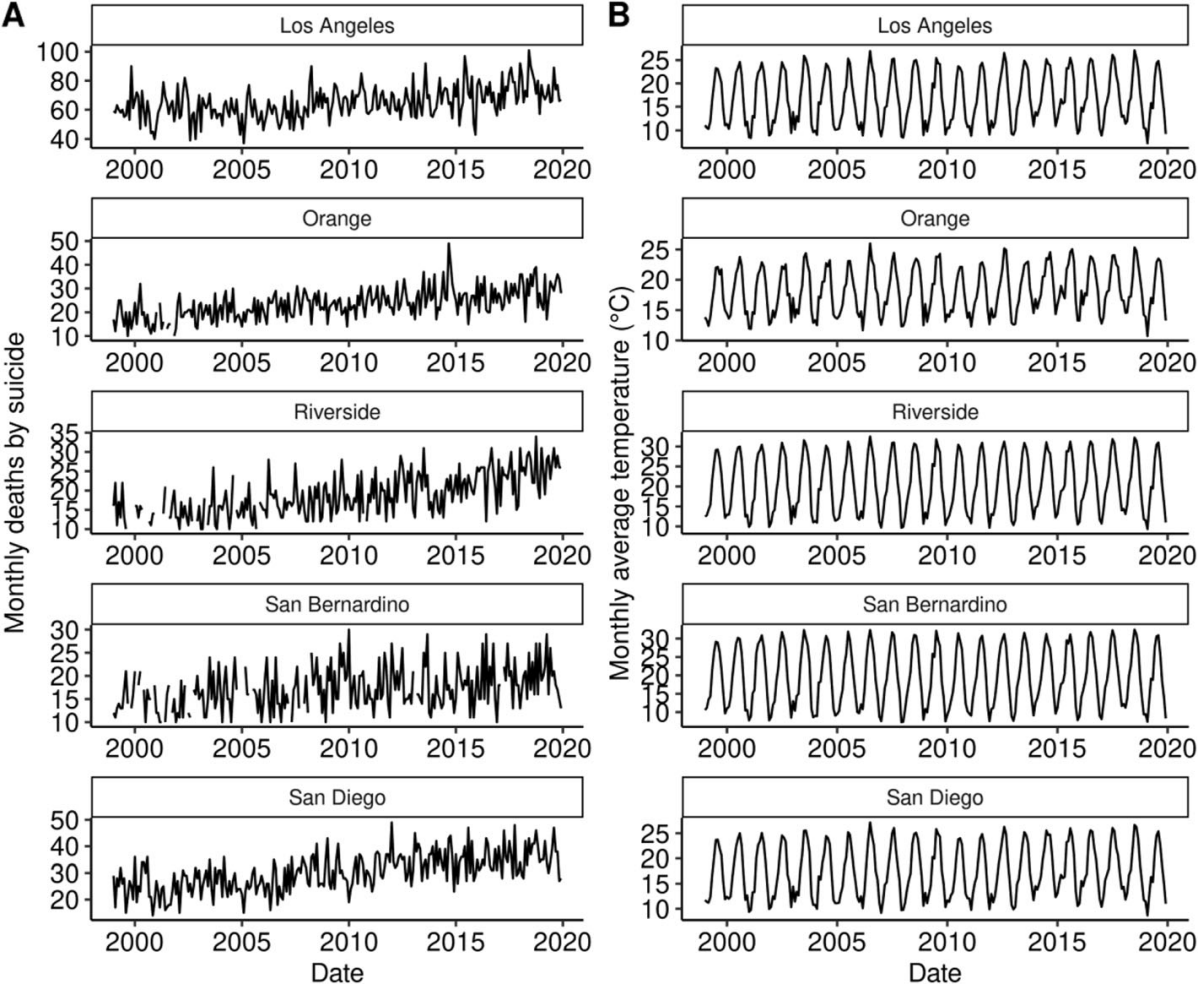
Monthly deaths by suicide and average monthly temperature in five urban California counties (1999–2019). Monthly counts of death by suicide smaller than 10 have been suppressed and are shown on the plot as missing values

**Fig. 2:**
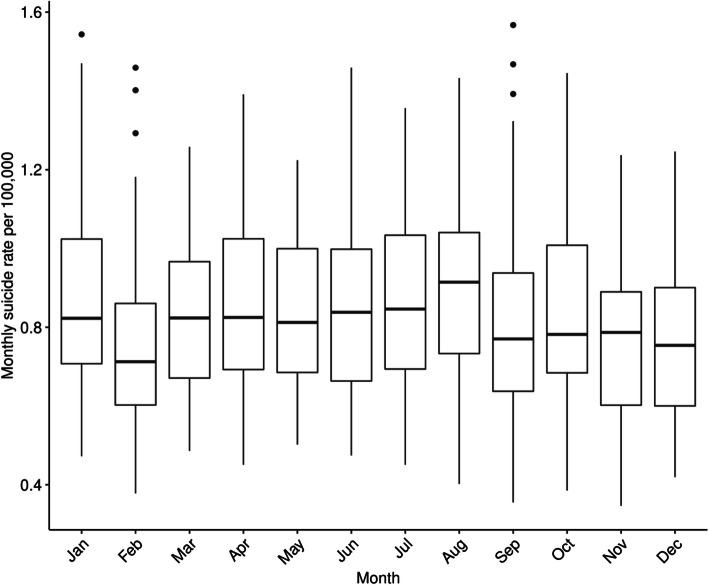
Tukey-style boxplot of seasonal variation in monthly suicide rate per 100,000 in five urban California counties (1999–2019)

Figure 1 visualizes the time series for monthly deaths by suicide and monthly average temperature by county. In most counties, a positive trend in the number of suicides recorded over time is evident. Table 1 summarizes the annual trends for both suicide rate per 100,000 and average temperature by county, while Fig. 2 summarizes the seasonality of suicide rates per 100,000.

**Table 1:** Annual suicide rate per 100,000 and annual average temperature (°C) in five urban California counties (1999–2019)

	Annual suicide rate per 100,000					Annual average temperature (°C)				
	LA	O	R	SB	SD	LA	O	R	SB	SD
**1999**	7.83	7.42	8.89	9.34	10.50	16.1	17.2	20.0	19.0	16.7
**2000**	7.22	7.60	8.79	8.85	11.04	16.6	17.8	20.4	19.6	17.3
**2001**	7.72	4.82	7.73	7.10	9.06	16.4	17.3	20.3	19.6	17.0
**2002**	7.56	8.25	8.00	8.17	10.72	16.4	17.4	20.2	19.4	16.9
**2003**	7.45	8.30	9.03	10.62	11.18	16.9	18.0	20.7	19.8	17.5
**2004**	7.12	8.67	9.32	8.74	10.61	16.5	17.8	20.1	19.1	17.0
**2005**	7.13	8.10	8.70	7.92	10.21	16.4	17.9	20.1	19.1	17.1
**2006**	6.89	9.17	10.14	9.78	9.84	16.6	18.1	20.3	19.4	17.5
**2007**	7.08	9.55	10.17	6.88	12.17	16.8	17.9	20.5	19.7	17.3
**2008**	8.32	9.81	9.67	9.68	12.08	17.0	18.3	20.6	19.4	17.6
**2009**	8.04	8.57	10.29	11.97	12.02	16.7	18.3	20.5	19.3	17.5
**2010**	8.24	9.45	8.58	10.49	11.44	15.9	17.3	19.8	18.7	16.6
**2011**	7.88	10.13	10.12	9.70	12.30	16.0	17.3	19.7	18.6	16.7
**2012**	7.74	9.75	11.76	10.80	12.88	17.2	18.3	20.9	20.0	17.7
**2013**	7.99	10.08	10.50	9.46	13.43	17.1	17.9	20.5	19.4	17.5
**2014**	8.15	11.55	9.71	9.29	12.96	18.2	19.4	21.5	20.6	18.8
**2015**	8.16	9.30	10.96	10.12	12.53	17.8	19.5	21.1	20.1	18.3
**2016**	8.34	10.11	11.93	10.74	12.55	17.6	18.9	21.2	20.1	18.2
**2017**	8.82	10.30	11.26	10.14	13.01	17.8	19.1	21.5	20.4	18.4
**2018**	9.40	11.78	12.67	10.94	13.44	17.5	18.8	21.2	20.2	18.2
**2019**	8.62	10.9	12.95	10.83	12.79	16.1	17.7	19.8	18.6	16.9

*LA* Los Angeles County, *O* Orange County, *R* Riverside County, *SB* San Bernardino County, *SD* San Diego County. Annual suicide rates were calculated from monthly county-level data, which exclude a small percentage of monthly counts suppressed due to small cell sizes in Orange, Riverside and San Bernardino Counties. Annual average temperature was calculated as the mean of all monthly average temperature values

**Fig. 3:**
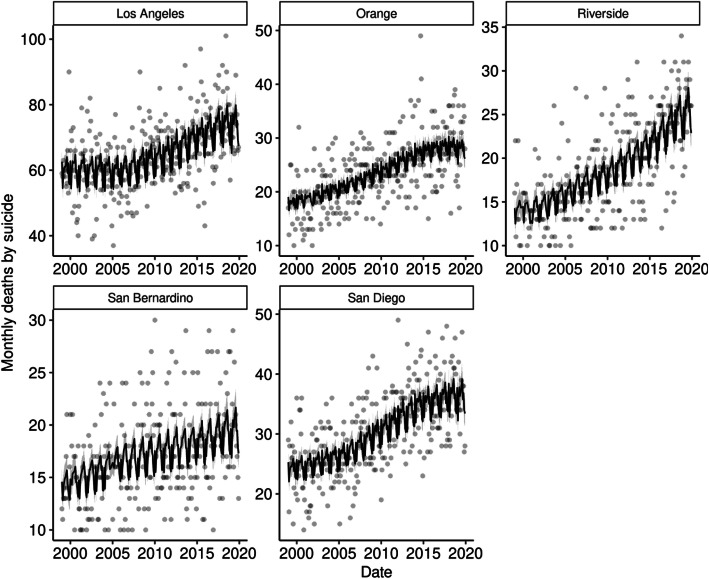
Fitted values of the negative binomial model of the relationship between average monthly temperature and monthly suicide rate in five urban California counties (1999–2019). The 95% confidence interval of the fitted values is shown in grey. Observed monthly values are indicated by grey dots

For all model formulations (baseline, average temperature, maximum temperature, minimum temperature), the negative binomial models (Table 2) demonstrated better relative model fit than the equivalent Poisson models (Supplementary Table 1), likely due to the presence of overdispersion in the model. Thus, only the negative binomial models will be further discussed. According to AIC, all three models incorporating temperature demonstrated a better relative fit than the baseline model (ΔAIC > 2). While the model with average temperature had the lowest AIC value, the other models demonstrated similar relative fit (ΔAIC_min_ < 2). The estimated coefficients were similar for all models: average temperature (IRR = 1.0082 per °C; 95% CI = 1.0025–1.0140), maximum temperature (IRR = 1.0069 per °C; 95% CI = 1.0021–1.0117), and minimum temperature (IRR = 1.0088 per °C; 95% CI = 1.0023–1.0153). Each model explained just over half of the total deviance (Table 2). The fitted values for the negative binomial model incorporating average temperature are shown in Fig. 3.

**Table 2:** Negative binomial model results for monthly suicide rate in five urban California counties (1999–2019)

	Baseline (Temperature Excluded)		Average Temperature		Maximum Temperature		Minimum Temperature	
	IRR	95% CI	IRR	95% CI	IRR	95% CI	IRR	95% CI
**Temperature**								
**Average**	–		1.0082	1.0025–1.0140	–		–	
**Maximum**	–		–		1.0069	1.0021–1.0117	–	
**Minimum**	–		–		–		1.0088	1.0023–1.0153
**Month**								
**January (ref.)**	–		–		–		–	
**February**	0.8759	0.8308–0.9234	0.8740	0.8292–0.9212	0.8742	0.8293–0.9214	0.8741	0.8292–0.9214
**March**	0.9608	0.9125–1.0117	0.9418	0.8930–0.9933	0.9421	0.8933–0.9936	0.9440	0.8952–0.9955
**April**	0.9980	0.9483–1.0504	0.9632	0.9103–1.0192	0.9638	0.9109–1.0198	0.9672	0.9146–1.0228
**May**	0.9912	0.9417–1.0433	0.9335	0.8740–0.9970	0.9382	0.8801–1.0001	0.9352	0.8748–0.9997
**June**	0.9944	0.9447–1.0467	0.9056	0.8338–0.9836	0.9121	0.8428–0.9872	0.9089	0.8359–0.9884
**July**	1.0154	0.9650–1.0683	0.9019	0.8189–0.9933	0.9129	0.8343–0.9988	0.9028	0.8165–0.9983
**August**	1.0416	0.9902–1.0956	0.9261	0.8415–1.0192	0.9359	0.8552–1.0242	0.9286	0.8414–1.0248
**September**	0.9570	0.9088–1.0078	0.8659	0.7942–0.9441	0.8726	0.8035–0.9477	0.8692	0.7961–0.9490
**October**	0.9821	0.9327–1.0341	0.9214	0.8610–0.9860	0.9254	0.8663–0.9886	0.9244	0.8635–0.9895
**November**	0.8979	0.8519–0.9464	0.8745	0.8274–0.9244	0.8753	0.8282–0.9251	0.8766	0.8295–0.9265
**December**	0.8855	0.8402–0.9332	0.8897	0.8442–0.9376	0.8902	0.8447–0.9381	0.8884	0.8431–0.9363
**Model Characteristics**								
**AIC**	7601.71		7595.17		7595.69		7596.27	
**% Deviance Explained**	50.84%		51.27%		51.22%		51.21%	

*AIC* Akaike information criterion, *CI* confidence interval, *IRR* incidence rate ratio

For all negative binomial models, Q-Q plots of the residual distributions indicated good model fit (Supplementary Fig. 1). Autocorrelation plots showed no evidence of residual autocorrelation. These plots for the negative binomial average temperature model are shown in Supplementary Fig. 2.

As a sensitivity analysis, the negative binomial models were re-fit after imputing the suppressed values (Supplementary Table 2). The estimated coefficients were similar to those estimated in the main analysis: average temperature (IRR = 1.0084 per °C; 95% CI = 1.0026–1.0142); maximum temperature (IRR = 1.0072 per °C; 95% CI = 1.0023–1.0121); and minimum temperature (IRR = 1.0086 per °C; 95% CI = 1.0020–1.0153).

In another sensitivity analysis, we added the county-level unemployment rate to the best-fit model (negative binomial model with average temperature), trying 7 different lags (0–6 months). In none of the 7 fitted models did the estimated IRR for average temperature change substantially due to the inclusion of the unemployment rate (range of values: 1.0085–1.0087 compared to 1.0082 per °C in the best-fit model).

## Discussion

This study assessed the association between temperature and suicide rates in five urban California counties over a 21-year period. We found that a 1 °C increase in temperature corresponded to a 0.82% (95% CI: 0.25–1.40%) increase in the expected suicide rate after accounting for both the county-level annual trend and monthly seasonality. Similar results were obtained when average temperature was substituted with maximum and minimum temperature, which is unsurprising given the close relationship between these measures. While the size of this effect is fairly small relative to other risk factors for suicide, ambient temperature is a population-wide exposure, meaning the cumulative effects could be large in the context of a warming climate.

These results add to a growing body of literature analyzing the association between suicide and temperature [11]. For example, a large analysis by Burke et al. (2018) of US counties and Mexican municipalities found a 0.7% increase in suicide rates per 1 °C increase in average monthly temperature in US counties and a 2.1% increase in Mexican municipalities [10]. This finding held in both warm and cool regions and there was no evidence that the adoption of air conditioning over time blunted this relationship. Using daily temperature and emergency room visit data from California, Basu et al. (2018) found that a 1 °C increase in apparent temperature (a combination of ambient temperature and humidity) was associated with a 1% increase in emergency room visits for self injury/suicide during the months of May to October [32].

Our study supports a causal link between ambient temperature and suicide rates in California after accounting for county-specific annual trends and monthly seasonality. There are many other predictors of suicide, such as unemployment, housing, mental health diagnoses, and crop failures, which were absent from our primary model [33, 34]. However, to be considered potential confounders of our effect of interest, these variables would have to be causes (or associated with causes) of ambient temperature. If anything, some of these predictors are plausibly *mediators* of the relationship between temperature and suicide, or possibly effect measure modifiers. Solar radiation may constitute a possible exception [6]. While some previous research on temperature and mortality has controlled for air pollution, this variable is more likely to be a mediator or effect measure modifier of the effect of temperature on mortality, rather than a confounder [35]. Altitude affects ambient temperature and has also been associated with suicide rates; however, it is unchanging over the study period and would be absorbed into the county-specific intercepts [34].

Our study had several limitations. As daily suicide data were not available, we were unable to examine the effects of daily temperature variation on suicide rates as some other studies have done [14, 32, 33]. Other limitations in the CDC WONDER dataset, particularly regarding the suppression of monthly mortality counts below 10, precluded an analysis stratified by age, sex, race, or other potentially relevant demographic factors. We were also unable to include rural counties in our analysis, where suicide tends to be more common [36]. Since the risk of suicide differs across demographic groups, sub-population analyses would add valuable insight [37]. The database also does not provide age-adjusted suicide rates for monthly data (which negate the effect of changing demographics in a population) due to the lack of a valid population. However, we do not believe that not accounting for demographic change constitutes a threat to our analysis as any temporal trend caused by a changing population structure over time would be captured by the term for the county-level annual trend.

Given the rising spectre of climate change, it is vital to further elucidate the causal mechanisms between temperature and suicide as well as the other mental health impacts of a changing climate [38]. Psychological research on the connection between heat and aggression reveal this relationship may arise from an increase in arousal, negative and hostile thoughts, and reduced cognitive function [38, 39]. It should be noted that much of the variation in monthly counts of suicide remained unexplained in our temperature-based analysis (Fig. 3). Further investigation into environmental, biological, and societal contexts that influence suicide is imperative for the development and implementation of preventative strategies and policies. Finally, it is important to recognize that stigma remains a major barrier to suicide prevention and the accurate measurements of trends in suicide.

## Conclusion & Future Directions

In our analysis of mortality data from five urban California counties over a 21-year period, we demonstrated a small but potentially important effect of increasing temperature on suicide rates after accounting for annual and seasonal trends in incidence. These results support the mounting evidence of a causal association between temperature and suicide. Climate change is set to exacerbate many existing public health issues, including mental health and suicide. Despite this, California’s Strategic Plan for Suicide Prevention for 2020–2025 did not explicitly include strategies to mitigate the impact of climate change on suicide [40]. Further research is required to illuminate the complex web of biological and social mechanisms mediating the relationship between temperature and suicide in order to inform the development of public health policy. Additionally, research to identify groups most at risk of climate-related harms is necessary as we confront the evolving effects of climate change.

## Supplementary Information

**Additional file 1: Supplementary Table 1.** Poisson model results for monthly suicide rate in five urban California counties (1999–2019). **Supplementary Table 2.** Negative binomial model results for monthly suicide rate in five urban California counties (1999–2019) after imputation of suppressed counts. **Supplementary Figure 1.** Quartile-quartile plots for the negative binomial models of monthly suicide rate. The straight line shows the expected distribution of the residuals. **Supplementary Figure 2.** Autocorrelation plots by county of the response residuals from the negative binomial model of the relationship between average monthly temperature and monthly deaths by suicide in five urban California counties (1999–2019). The blue dashed lines indicate lags at which the autocorrelation is statistically significantly different from 0.

## Data Availability

We used datasets obtained from the following databases, as described in the Methods: Underlying Cause of Death database of CDC WONDER (https://wonder.cdc.gov/ucd-icd10.html), nCLIMDIV database from the National Oceanic and Atmospheric Administration (date of data release: 2021-02-04) (https://www.ncei.noaa.gov/access/metadata/landing-page/bin/iso?id=gov.noaa.ncdc:C00005), U.S. Population Data - 1969-2019 database from the National Cancer Institute’s Surveillance Epidemiology, and End Results Program (https://seer.cancer.gov/popdata/download.html), and the Local Area Unemployment Statistics database from the State of California’s Employment Development Department (https://data.edd.ca.gov/Labor-Force-and-Unemployment-Rates/Local-Area-Unemployment-Statistics-LAUS-/e6gw-gvii). All datasets used in this manuscript are publicly available at the URLs given. Additionally, copies of all datasets and the R code necessary to reproduce the results presented in this manuscript are publicly available in the following GitHub repository: https://github.com/jeanpaulrsoucy/california-counties-suicide-and-temperature.

## Abbreviations

IRR: Incidence Rate Ratio
CI: Confidence Interval
CDC: Centers for Disease Control and Prevention
WONDER: Wide-ranging ONline Data for Epidemiologic Research
AIC: Akaike information criterion
Q-Q: Quantile-Quantile
