# Supplementary material for: The effect of average temperature on suicide rates in five urban California counties, 1999–⁠2019: an ecological time series analysis
Source: BMC Public Health. 2021 May 25;21:974. doi: 10.1186/s12889-021-11001-6 (PMC8147045; doi:10.1186/s12889-021-11001-6)
Supplement: Supplementary file 1 — Additional file 1: Supplementary Table 1. Poisson model results for monthly suicide rate in five urban California counties (1999–2019). Supplementary Table 2. Negative binomial model results for monthly suicide rate in five urban California counties (1999–2019) after imputation of suppressed counts. Supplementary Figure 1. Quartile-quartile plots for the negative binomial models of monthly suicide rate. The straight line shows the expected distribution of the residuals. Supplementary Figure 2. Autocorrelation plots by county of the response residuals from the negative binomial model of the relationship between average monthly temperature and monthly deaths by suicide in five urban California counties (1999–2019). The blue dashed lines indicate lags at which the autocorrelation is statistically significantly different from 0. [file 12889_2021_11001_MOESM1_ESM.docx]

**Supplementary Table 1.** Poisson model results for monthly suicide rate in five urban California counties (1999–2019).

|  | **Baseline (Temperature Excluded)** | | **Average Temperature** | | **Maximum Temperature** | | **Minimum Temperature** | |
| --- | --- | --- | --- | --- | --- | --- | --- | --- |
|  | **IRR** | **95% CI** | **IRR** | **95% CI** | **IRR** | **95% CI** | **IRR** | **95% CI** |
| **Temperature** | | | | | | | | |
| **Average** | - | | 1.0083 | 1.0029-1.0139 | - | | - | |
| **Maximum** | - | | - | | 1.0070 | 1.0024-1.0116 | - | |
| **Minimum** | - | | - | | - | | 1.0088 | 1.0026-1.0151 |
| **Month** | | | | | | | | |
| **January (ref.)** | - | | - | | - | | - | |
| **February** | 0.8772 | 0.8345-0.9220 | 0.8753 | 0.8327-0.9201 | 0.8754 | 0.8328-0.9202 | 0.8754 | 0.8328-0.9202 |
| **March** | 0.9607 | 0.9151-1.0085 | 0.9415 | 0.8953-0.9902 | 0.9418 | 0.8955-0.9904 | 0.9440 | 0.8978-0.9926 |
| **April** | 0.9974 | 0.9506-1.0466 | 0.9624 | 0.9123-1.0152 | 0.9628 | 0.9128-1.0156 | 0.9668 | 0.9171-1.0193 |
| **May** | 0.9925 | 0.9458-1.0415 | 0.9342 | 0.8777-0.9944 | 0.9389 | 0.8838-0.9975 | 0.9366 | 0.8793-0.9977 |
| **June** | 0.9950 | 0.9481-1.0442 | 0.9053 | 0.8369-0.9792 | 0.9117 | 0.8457-0.9829 | 0.9096 | 0.8400-0.9849 |
| **July** | 1.0162 | 0.9688-1.0660 | 0.9015 | 0.8223-0.9883 | 0.9123 | 0.8374-0.9939 | 0.9036 | 0.8212-0.9943 |
| **August** | 1.0413 | 0.9930-1.0920 | 0.9247 | 0.8441-1.0130 | 0.9343 | 0.8575-1.0180 | 0.9285 | 0.8454-1.0198 |
| **September** | 0.9579 | 0.9124-1.0057 | 0.8657 | 0.7973-0.9399 | 0.8723 | 0.8064-0.9436 | 0.8700 | 0.8003-0.9458 |
| **October** | 0.9821 | 0.9355-1.0309 | 0.9207 | 0.8634-0.9819 | 0.9246 | 0.8684-0.9845 | 0.9245 | 0.8666-0.9861 |
| **November** | 0.8975 | 0.8540-0.9431 | 0.8739 | 0.8292-0.9211 | 0.8747 | 0.8300-0.9217 | 0.8763 | 0.8316-0.9234 |
| **December** | 0.8846 | 0.8418-0.9296 | 0.8889 | 0.8458-0.9342 | 0.8894 | 0.8463-0.9348 | 0.8877 | 0.8446-0.9328 |
| **Model Characteristics** | | | | | | | | |
| **AIC** | 7605.69 | | 7598.56 | | 7598.69 | | 7599.77 | |
| **% Deviance Explained** | 51.62% | | 51.97% | | 51.97% | | 51.93% | |

AIC: Akaike information criterion; CI: Confidence interval; IRR: Incidence rate ratio.

**Supplementary Table 2.** Negative binomial model results for monthly suicide rate in five urban California counties (1999–2019) after imputation of suppressed counts.

|  | **Baseline (Temperature Excluded)** | | **Average Temperature** | | **Maximum Temperature** | | **Minimum Temperature** | |
| --- | --- | --- | --- | --- | --- | --- | --- | --- |
|  | **IRR** | **95% CI** | **IRR** | **95% CI** | **IRR** | **95% CI** | **IRR** | **95% CI** |
| **Temperature** | | | | | | | | |
| **Average** | - | | 1.0084 | 1.0026-1.0142 | - | | - | |
| **Maximum** | - | | - | | 1.0072 | 1.0023-1.0121 | - | |
| **Minimum** | - | | - | | - | | 1.0086 | 1.0020-1.0153 |
| **Month** | | | | | | | | |
| **January (ref.)** | - | | - | | - | | - | |
| **February** | 0.8666 | 0.8209-0.9149 | 0.8649 | 0.8194-0.9129 | 0.8651 | 0.8195-0.9131 | 0.8650 | 0.8194-0.9131 |
| **March** | 0.9538 | 0.9045-1.0058 | 0.9345 | 0.8847-0.9870 | 0.9343 | 0.8846-0.9869 | 0.9373 | 0.8876-0.9899 |
| **April** | 0.9949 | 0.9439-1.0486 | 0.9594 | 0.9053-1.0168 | 0.9593 | 0.9052-1.0165 | 0.9646 | 0.9107-1.0217 |
| **May** | 0.9849 | 0.9343-1.0382 | 0.9260 | 0.8654-0.9908 | 0.9296 | 0.8705-0.9927 | 0.9297 | 0.8681-0.9957 |
| **June** | 0.9841 | 0.9336-1.0374 | 0.8944 | 0.8219-0.9732 | 0.8992 | 0.8292-0.9750 | 0.9007 | 0.8268-0.9813 |
| **July** | 1.0149 | 0.9631-1.0695 | 0.8994 | 0.8150-0.9925 | 0.9083 | 0.8285-0.9958 | 0.9041 | 0.8160-1.0017 |
| **August** | 1.0418 | 0.9889-1.0975 | 0.9241 | 0.8381-1.0190 | 0.9319 | 0.8499-1.0217 | 0.9305 | 0.8414-1.0290 |
| **September** | 0.9488 | 0.8997-1.0006 | 0.8566 | 0.7842-0.9357 | 0.8616 | 0.7919-0.9375 | 0.8629 | 0.7888-0.9439 |
| **October** | 0.9651 | 0.9153-1.0176 | 0.9045 | 0.8440-0.9693 | 0.9073 | 0.8481-0.9707 | 0.9094 | 0.8482-0.9749 |
| **November** | 0.8886 | 0.8420-0.9378 | 0.8654 | 0.8176-0.9159 | 0.8657 | 0.8180-0.9161 | 0.8682 | 0.8204-0.9188 |
| **December** | 0.8837 | 0.8373-0.9327 | 0.8880 | 0.8414-0.9372 | 0.8887 | 0.8420-0.9379 | 0.8866 | 0.8401-0.9357 |
| **Model Characteristics** | | | | | | | | |
| **AIC** | 7924.96 | | 7919.00 | | 7918.77 | | 7920.44 | |
| **% Deviance Explained** | 46.43% | | 46.80% | | 46.81% | | 46.73% | |

AIC: Akaike information criterion; CI: Confidence interval; IRR: Incidence rate ratio.

**Supplementary Figure 1.** Quartile-quartile plots for the negative binomial models of monthly suicide rate. The straight line shows the expected distribution of the residuals.


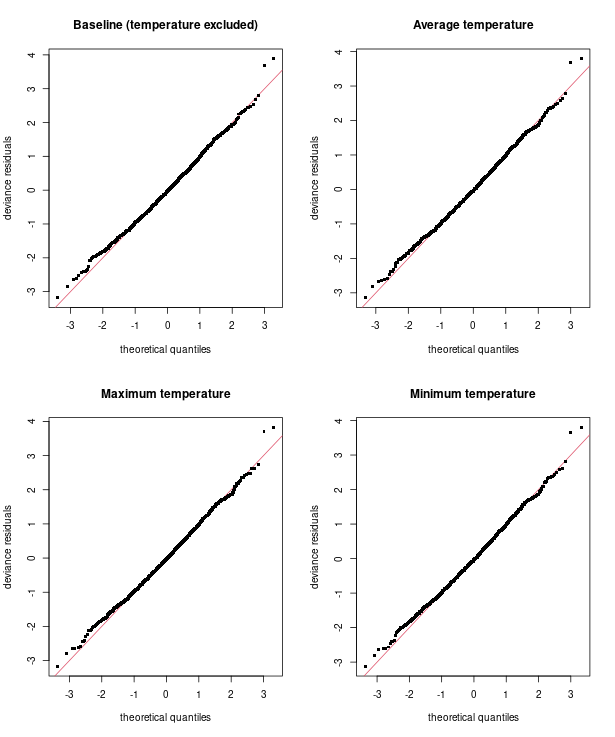


**Supplementary Figure 2.** Autocorrelation plots by county of the response residuals from the negative binomial model of the relationship between average monthly temperature and monthly deaths by suicide in five urban California counties (1999–2019). The blue dashed lines indicate lags at which the autocorrelation is statistically significantly different from 0.

**
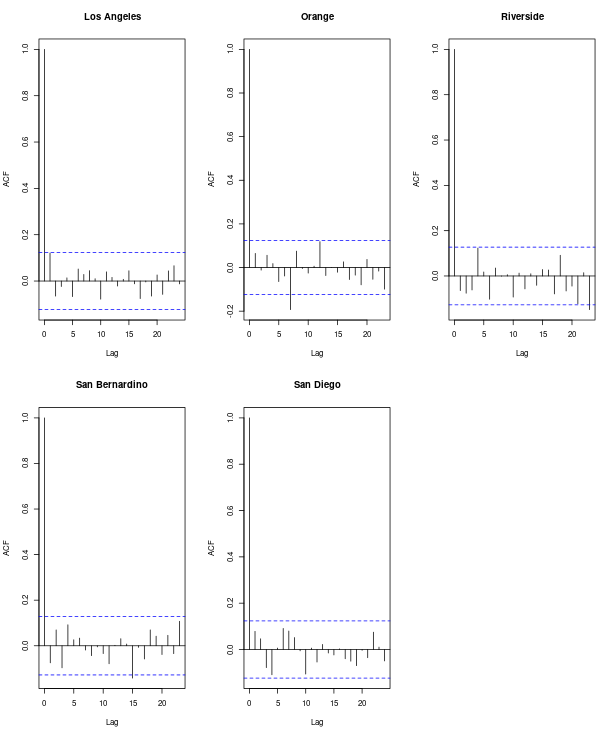
**
